# Design, implementation, and evaluation of IIIDDS: a structured WhatsApp case-discussion curriculum in undergraduate radiology education

**DOI:** 10.3389/fmed.2026.1789785

**Published:** 2026-04-10

**Authors:** Mustafa Mohammed Al-Mashhadani, Sameer Asim Khan, Varshini Tamil Selvan, Rania Atef Soued

**Affiliations:** 1 College of Medicine, Mohammed Bin Rashid University of Medicine and Health Sciences, Dubai Health, Dubai, United Arab Emirates; 2 Pediatric Department, Al Jalila Children's Specialty Hospital, Dubai Health, Dubai, United Arab Emirates; 3 Radiology Department, Emirates Specialty Hospital, Dubai, United Arab Emirates

**Keywords:** case-based learning, digital medical education, instructional design, medical education, mobile applications, radiology

## Abstract

**Background:**

Instant-messaging platforms like WhatsApp may be a useful and scalable adjunct to undergraduate medical education, particularly when it comes to image-rich disciplines like radiology. We developed a concise, repeatable case-discussion framework for large-scale instant-messaging group chats (IIIDDS: Indication, Imaging, Interpretation, Differentials, Discussion, and Safety-net) to support a student-led, faculty-moderated case-discussion group model.

**Objective:**

To explore learner perceptions of a student-led, faculty-moderated radiology WhatsApp group and its structured IIIDDS case-discussion approach between preclinical and clinical undergraduate medical students.

**Methods:**

A Checklist for Reporting Results of Internet E-Surveys (CHERRIES) informed exploratory cross-sectional survey was distributed to the Radiology Club WhatsApp members via email and WhatsApp. The survey explored perceived usability, educational value, learning environment, and challenges of the platform and IIIDDS model. Descriptive analyses and subgroup comparisons were performed between medical students with and without clinical rotation experience.

**Results:**

In total, 128 responses were evaluated in the analysis. Overall comfort with WhatsApp was high (mean 4.50/5). Participants commonly valued shared radiology cases (82.0%), annotated images (76.6%), and faculty guidance (70.3%). Clinical students reported being more comfortable using WhatsApp (mean 4.75 vs. 4.32; *p* = 0.006) and a stronger perception of a safe learning environment (mean 3.75 vs. 3.33; *p* = 0.031) compared to preclinical students. Reported challenges included overwhelming message volume (68.0%) and difficulty keeping up with discussions (61.7%).

**Discussion:**

A structured, student-led, faculty-moderated instant-messaging-based case discussion model was perceived as an accessible and credible adjunct for undergraduate radiology education. Providing a structured case presentation framework and a platform for accessible multimedia sharing was perceived to support reliable and constructive case discussions by combining structured discussion with faculty moderation. Findings suggest practical design priorities for similar implementations, including faculty moderation, structure, and strategies to reduce message-overload.

## 1 Introduction

Smartphones have revolutionized the world in their ability to provide quick and reliable communication and access to the internet. The accessibility and ownership of smartphones has accelerated to becoming a staple of daily life, impacting the learning environment of medical students throughout the world (1, 2). Smartphones have substantially influenced medical education by enabling rapid access to learning resources while facilitating communication between students and educators (1, 3). The influence of smartphones on medical students is particularly compounded by recent studies that report that the majority of medical students from different parts of the world own smartphones and are thus impacted by them (2, 4–6). Importantly for this study, the ability to learn via mobile devices and social media has been shown to enhance engagement, feedback, and communication between students and educators (7). In particular, WhatsApp, a free cross-platform messaging service available on mobile devices and owned by the company Meta Platforms, Inc. (Menlo Park, CA, United States), has demonstrated promising advantages in delivering electronic-based medical education (8, 9). A scoping review by Coleman and O'Connor argued that WhatsApp can be an effective and convenient educational modality in medicine, particularly when an educational curriculum is developed tailored to the group chats (8).

Radiology education is particularly unique in its challenges due to its reliance on the interpretation of complex and sometimes difficult to comprehend images while requiring the usage of clinical reasoning skills (10, 11). Recent studies have demonstrated positive usage of the WhatsApp platform by educators to provide adjunctive radiology teaching to medical trainees (12, 13). Particularly, an instant-messaging platform like WhatsApp may enable real-time case sharing, interactive discussions, and a place for readily sharing media like images (13). Its convenient access, as well as the capability to magnify images, have been reported as advantages of the platform for imaging education (14).

While WhatsApp and other instant messaging platforms have shown potential in medical education, existing literature focuses on postgraduate training or specific specialities like dental or pediatric radiology (12–14). While WhatsApp has been used in medical education, there is limited published work describing a structured, faculty-moderated WhatsApp case-discussion model for undergraduate radiology learning, particularly amongst a group of students not particularly specializing or intending to specialize in radiology. Moreover, less attention has been given to how a structured learning framework may be embedded within an instant-messaging platform to support radiology medical education. This study aims to address these gaps by exploring learner perceptions regarding the strengths and limitations of an existing radiology group chat structure amongst undergraduate medical students. This study also seeks to inform about practices for the development of large internet-based group chats for medical educational purposes. This article describes an educational innovation developed over several years tailored to the realities of institutional radiological education and reports early evaluation data focused on feasibility, acceptability, and learner perceptions rather than reporting objective learning outcomes.

## 2 Pedagogical framework

The MBRU Radiology Club’s main educational modality was designed as a structured, case-based learning community delivered via the instant-messaging platform WhatsApp. The pedagogical rationale was that WhatsApp’s accessibility and ease of use can enable mass-participation with an instructional design that supports coherent discussion rather than fragmented conversation (15). The IIIDDS model (Indication, Imaging, Interpretation, Differentials, Discussion, and Safety-net) was developed as a concise and repeatable script for case discussions that can be used by novice and experienced presenters. The framework was designed to function as a scaffold that organizes group chat interactions into predictable stages, supporting learners to move from clinical context (Indication) to investigation selection (Imaging/Findings), pattern recognition and explanation (Interpretation), hypothesis generation (Differentials), and synthesis/management reasoning (Discussion). This staging is consistent with the premise of cognitive load theory that well-structured learning tasks can reduce extraneous load and ambiguity, particularly in complex, image-rich domains such as radiology (16, 17). The model and platform also allow for repeated exposure to a variety of cases (1–2 discussions weekly), a strategy shown to help in learner retention, with frequent opportunities for retrieval, feedback, and refinement of reasoning across different levels of case complexity (18, 19). Radiology faculty moderation and review of cases before presentation were incorporated to maintain credibility and validity of information before dissemination while allowing for immediate faculty feedback, which may help in reducing the risks of misinformation that may be propagated via social mobile applications (20, 21). Anxiety and fear of making mistakes may also be challenges in promoting participation amongst large group chats (22). The inclusion of a dedicated “Safety-net” stage, the ability for participants to contribute privately to presenters, and a points system that awards partial credit to incorrect answers were intended to normalize uncertainty and encourage participation even amongst novice learners. At the competency level, the model was designed to supplement institutional undergraduate medical learning objectives by reinforcing skills relevant to radiology and general clinical reasoning, selecting appropriate investigations, interpreting imaging/findings, generating and narrowing differential diagnoses, and discussing management implications are all important skills for medical students and future physicians (23–25). In the local UAE context, these outcomes are emphasized in curricula with competency-based approaches that focus on clinical reasoning, patient safety, communication (including written/electronic communication), professionalism and confidentiality, collaboration, and lifelong learning (26).

Moreover, the cases selected for presentation vary seasonally depending on student curriculum and their progression with the M.D. program. Radiology faculty-moderation allows the group chat’s cases to be timely tailored to the College of Medicine’s curriculum, concentrating cases based off current student experiences while allowing senior students to refresh and recall prior learning. For example, first-year medical students at MBRU may learn about limb and spine anatomy in their first semester, while third-year medical students would be exposed to gastrointestinal anatomy at the same time. This leads to the curriculum focusing on limb and spine imaging while also involving more senior students with gastrointestinal imaging at the same time. As students progress through medical school, they are exposed to timely case-presentations and the ability for repetitive recall of past topics.

## 3 Learning environment

### 3.1 Study setting and context

**Figure 1:**
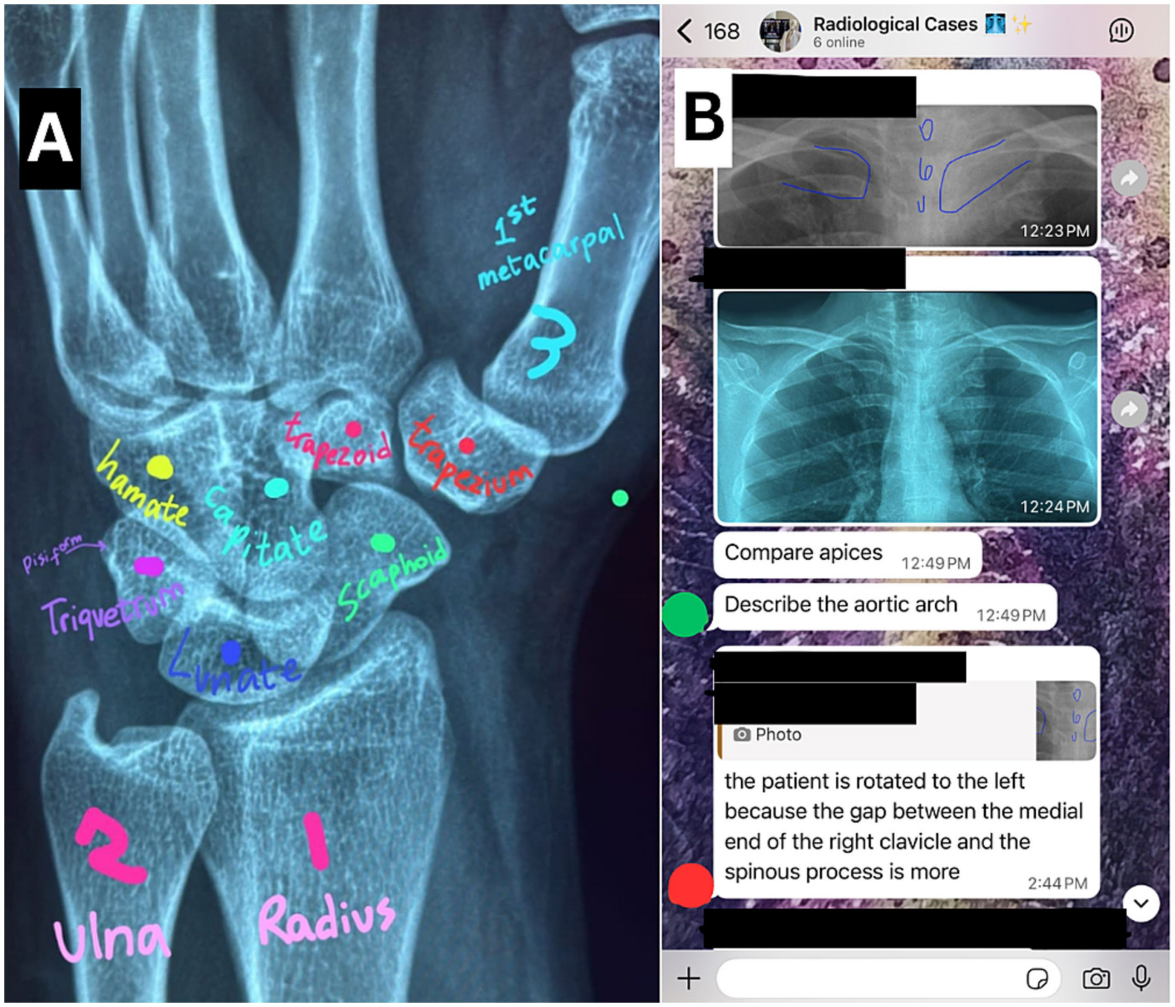
**(A)** A wrist radiograph annotated solely on the WhatsApp platform by a student, without the loss of image resolution or the requirements of any application add-ons, shared on the MBRU Radiology Club group chat during a discussion. Image reproduced with permission courtesy of Syeda Emaan Hussain. **(B)** A case discussion showcasing WhatsApp’s ease of use, access, and integration with annotation tools (blue markings on the radiograph). Note that the students’ (red) answer regarding the patient being rotated to the left was wrong, which was immediately corrected by an attending radiologist (green). The screenshot is shared with permission.

This study was conducted amongst undergraduate medical students enrolled or graduated from the Doctor of Medicine (M.D.) program in the College of Medicine (CoM) at the Mohammed Bin Rashid University of Medicine and Health Sciences (MBRU), Dubai Health, Dubai, United Arab Emirates (27). MBRU is a semi-public medical and health-sciences based university with four academic units, including the College of Medicine, launched in January 2016 (27–30). The MBRU Radiology club group chat was established on July 30th, 2020, as an online platform for radiological discussions in response to in-person learning restrictions due to the COVID-19 pandemic, with all MBRU M.D. students eligible to join the club’s WhatsApp group chat. The WhatsApp platform was chosen for the club due to its ease of use and popularity amongst MBRU students and faculty. The WhatsApp platform allows for the sharing of files up to 2GBs in size with compression features for multiple images or videos, which allows for high resolution images to be shared reliably on the platform (31). Since the size of a typical chest x-ray may be as large as 20 MBs, the WhatsApp platform allows for the sharing of high-resolution images that can be magnified and annotated without losing image quality, an important aspect to consider for an image-heavy field like radiology (see Figure 1) (32, 33). The MBRU Radiology Club WhatsApp group hosts 248 members as of March 17, 2026, including M.D. students (from years 1 through 6) and M.D. graduates (also called alumni). The club (including its events and the WhatsApp group chat) is led and organized by students actively enrolled in the MBRU M.D. program. The club holds case discussions 1–2 times weekly presented by students, alumni, or faculty who curate cases from online sources and/or their clinical experiences, moderated by an attending radiologist at the CoM while strictly ensuring de-identification of patient data for all cases (34). The cases presented are designed to closely align and supplement institutional undergraduate medical learning objectives with case complexities varying from basic concepts for new learners to more complex cases for intern students. Participants are encouraged to participate via two leaderboards, one with a point system for participation (1 point per correct answer, 0.25 points for an incorrect answer), and one leaderboard for the number of cases presented by a member, resetting at the end of every academic semester. The format of case discussions varies; however, a common and standardized framework developed by the MBRU Radiology Club involves the Indication, Imaging, Interpretation, Differentials, Discussion, and Safety-net (IIIDDS) model.

### 3.2 Learning objectives

The MBRU Radiology Club’s educational model was designed to support the following learning objectives for undergraduate medical students. By engaging with structured IIIDDS case discussions, learners are expected to (i) identify the clinical indications and contextual features relevant to imaging decisions; (ii) select and describe appropriate imaging modalities relevant to clinical presentations; (iii) interpret medical imaging via a structured and systematic approach; (iv) generate and refine a differential diagnosis list based on imaging and clinical findings; (v) discuss clinically relevant implications, including management considerations and communication of findings; (vi) explore patient outcomes and summarize case-discussions/presentations.

### 3.3 Pedagogical format: the IIIDDS case-discussion framework

**Figure 2:**
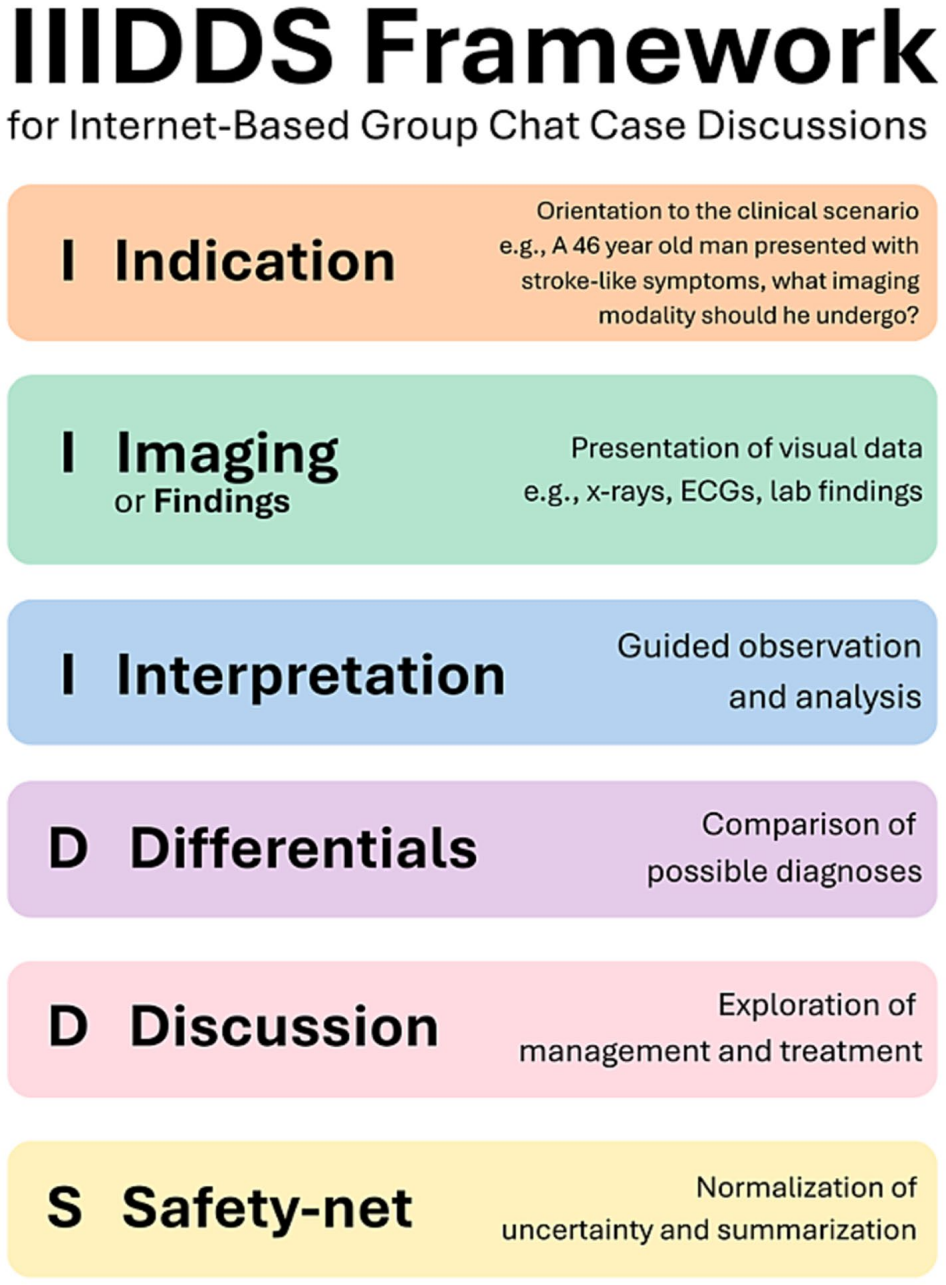
Visualization of the 6 steps of the IIIDDS framework for online group chat educational case-presentations used by the MBRU Radiology Club.

The IIIDDS model is scripted case-discussion framework for case-based teaching developed by students and radiology faculty at the College of Medicine and reworked since 2020. Before structuring the IIIDDS model, each discussion starts firstly with curation of the case, ensuring that presenters gather appropriate and valid information about the case, and anonymizing any patient data, regardless of if the case was obtained from clinical practice or online. The MBRU Radiology Club group chat employs experienced students in the role of “academic leads,” helping case presenters with the case’s academic information and in implementing the IIIDDS. Additionally, all cases are reviewed by an attending radiologist before presentation, ensuring valid and reliable information before dissemination. Following approval by an attending radiologist, presenters may then present, assisted throughout all steps by the academic leads and attending radiologists. An implementation checklist for the IIIDDS model is provided in Supplementary material S1 for replication. The IIIDDS model incorporates the following six steps, as illustrated in Figure 2:

1. *Indication*: Providing a short case representation (1–2 sentences, e.g., a 54-year-old man presents to the emergency department with leg pain following a fall from his roof) and asking what imaging modalities or investigations would be initially most appropriate (e.g., x-ray, CT polytrauma, E-FAST, arterial blood gas, etc.).
2. *Imaging*: The presenter will then display a radiologic image and ask for an interpretation (bonus points are given to students who ask for other radiological image views when applicable). If the case is not in radiology, this part can be replaced with “*Findings*,” displaying other relevant images or information like electrocardiographs (ECGs), physical examination findings, laboratory findings, or the results of whatever medical investigations are applicable.
3. *Interpretation*: A general back and forth case discussion occurs between the presenter and engaging students highlighting the pathologies of the case and relevant radiological learning points.
4. *Differentials*: Differential diagnoses are provided by the participants, and the case presenter helps participants rule in and out the differential diagnoses. Participants may ask for more investigations to help rule in or out diagnoses.
5. *Discussion*: Once a definitive diagnosis is established, a general discussion regarding the management and treatment of the patient is initiated.
6. *Safety-net*: The presenters are encouraged to safety-net by asking if any participants have any questions and answering them. At this point, further case interpretation is usually provided by an attending radiologist if not provided earlier, and summarization of the case occurs.

## 4 Results to date

### 4.1 Study design and enrolment

This study was a closed cross-sectional survey of members of the MBRU Radiology Club WhatsApp group chat. Inclusion criteria were: (i) being an MBRU M.D. student or alumnus, (ii) membership in the MBRU Radiology Club WhatsApp group, and (iii) at least one academic year of exposure to the group before survey participation. Exclusion criteria were: (i) current first-year M.D. students, as they had not yet completed one academic year of group exposure, (ii) individuals who were not current or former MBRU M.D. students, (iii) members with less than one academic year of exposure to the WhatsApp group, and (iv) investigator-authors involved in the study, due to a potential conflict of interest. Responses from eligible students and alumni of all ages and genders were included in the analysis. At the time of eligible responder estimation on June 1, 2025 (the end of the 2024–2025 academic year), the MBRU Radiology Club WhatsApp group had an estimated eligible survey population of 180 members. Eligibility was determined through administrative review of group membership based on the study inclusion and exclusion criteria, including the requirement for at least one academic year of group exposure. This denominator was used for sample-size estimation and response-rate calculation. A sample size calculation using the Raosoft sample size calculator yielded an approximate sample size between 112 to 123 participants for a confidence interval of 95% and a margin of error of 5% amongst a response distribution ranging from 50 to 75% (35).

The online questionnaire was made and distributed by utilizing the Microsoft Forms software, a web-based tool used for online questionnaires that can be used to prevent individuals from responding more than once (36). The online questionnaire was disseminated via WhatsApp and email. The first entry was received on September 8th, 2025, and the last on November 2nd, 2025, encompassing approximately 8 weeks. The survey items were created with the oversight of an expert academic radiologist (RS) involved with moderating the group chat by following the technology acceptance model (TAM) and the theory of planned behaviour (TPB) to assess perceptions of usefulness and ease-of-use of the group chat as a learning tool and to capture subjective norms with perceived behavioural patterns, respectively (37–41). The survey was designed to capture and evaluate undergraduate medical student views regarding the group chat’s educational usefulness, ease of use, and overall satisfaction. These outcomes were assessed via the post-intervention (IIIDDS) survey capturing subjective learner reactions, perceived value, and self-reported impact on confidence, consistent with early-stage program evaluation (42). No objective measures of educational competence were collected in this study (e.g., examination scores or pre/post intervention testing).

The methodology of this study and survey were designed to adhere to the Checklist for Reporting Results of Internet E-Surveys (CHERRIES) (43). The survey was sent to students via email and WhatsApp on multiple occasions. To ensure no repetition of responses, participants had to sign in with their institutional email address (Dubai Health) on Microsoft Forms, and each account was limited to one response which may be saved and revised; however, the investigators of this study could not see student identifiers (like names, email addresses, and identification numbers), ensuring anonymity of the users.

The survey utilized 18 5-point Likert scale questions and 5 multiple choice questions across 8 pages to collect the data used for the analysis. The first section collected basic demographic data about the participants and confirmed membership status (inclusion criteria) in the radiology club WhatsApp group chat while gaining consent for participation in the study. The second section evaluated the general WhatsApp usability habit of participants. Section 3 evaluated the usage pattern of the club’s WhatsApp group chat. Section 4 evaluated the effectiveness of the WhatsApp group in the radiological learning of participants. Section 5 evaluated the participation of the student in the WhatsApp group. Section 6 evaluated the reliability and validity of the WhatsApp group chat in terms of perceptions about information accuracy and in achieving undergraduate medical learning objectives. Section 7 asked about the positive strategies employed by the MBRU Radiology Club WhatsApp group, and section 8 evaluated pitfalls and the negative perceptions of the group chat.

### 4.2 Data analysis

This study employed descriptive statistics to describe perceived usefulness, ease of use, attitudes, behavioural intentions, and educational outcomes in the context of the MBRU Radiology Club WhatsApp group chat. Data analysis was conducted using the SPSS software (version 29.0.0.0; IBM Corp), and data visualization was conducted with the R programming software (R version 4.4.1; R Foundation for Statistical Computing). Descriptive analysis involved obtaining the mean 5-point Likert scale value and its standard deviation (SD) as well as tabulating variable frequencies. A sub-group analysis between preclinical (years 2 and 3) and clinical (years 4, 5, 6 and alumni) members was performed with the independent samples t-test analysis to determine if differences between members with and without clinical exposure were significant. Prior to the *t*-test, Levene’s test was used to assess if equal variances were met. A Cohen *d* test was performed to evaluate the extent of differences between the groups. The significance value was set at *p* < 0.050.

### 4.3 Ethical considerations

This study successfully received institutional review board (IRB) ethical approval from the Dubai Scientific Research Ethics Committee (DSREC) (approval identification number: MBRU IRB-2024-798). Participants were informed of the purpose of the study and written consent to participate in the research was obtained from the online survey. Participation was completely voluntary, and no personal identifiers of the participants was gathered by the study team. The investigators of this study who are involved in the MBRU Radiology Club WhatsApp group chat also did not fill out the survey due to a conflict of interest potentially biasing the results. The data was also anonymized, kept confidential, and available only to the study investigators within secured Dubai Health servers.

### 4.4 Student perceptions

A total of 154 responses were received. After excluding individuals who self-reported being part of the exclusion criteria (*n* = 26), 128 responses were included as part of this analysis (71.1% response rate). The 26 exclusions reflect post-response self-reported eligibility screening and reflect responses from individuals who were not part of the administratively estimated eligible survey population of 180 members. The results indicate that students especially valued platform features involving case sharing (82.0%, 105/128), ease of access (78.1%, 100/128), annotated images (76.6%, 98/128), and exposure to a variety of cases (72.7%, 93/128). Students reported moderately positive perceptions of the group’s educational effectiveness (mean 3.82/5, SD 0.86), and reported strong alignment with institutional teaching methods and learning outcomes (mean 4.31/5, SD 0.77). Students perceived the club group chat as highly reliable and credible (mean 4.41/5, SD 0.76), which may be attributed to the faculty-moderated model of the group chat as 70.3% (90/128) of participants cited faculty guidance as a positive factor of the club. Clinical students reported significantly greater comfort utilizing the WhatsApp platform in general (mean 4.75, SD 0.65 vs. mean 4.32, SD 1.09; *p* = 0.006) and a greater perception of a safe learning environment (mean 3.75, SD 1.04 vs. mean 3.33, SD 1.11; *p* = 0.031) compared with preclinical students. No other variables demonstrated significant differences between preclinical and clinical students.

In general, students reported a high usage of WhatsApp (mean 4.56/5, SD 0.86), where a score of 5 represented using WhatsApp multiple times a day. When asked about the multimedia and resource accessibility of the WhatsApp platform, students reported a high rating (mean 4.45/5, SD 0.63). Respondents reported moderately positive perceptions regarding their comfort learning from WhatsApp in general and the club’s group chat (mean 3.88/5, SD 1.12 and mean 3.82/5, SD 1.01 respectively).

Survey respondents reported the club’s teaching methods marginally boosted confidence in academic class participation (mean 3.30/5, SD 1.19) and examinations (mean 3.35/5, SD 1.13). Clinical-year students reported that the group chat moderately enhanced their clinical performance (mean 3.70/5, SD 1.20), while slightly beneficial for board examinations (mean 3.41/5, SD 0.91). Respondents cited being somewhat comfortable participating in discussions (mean 3.34/5, SD 1.05) and found them moderately enjoyable (mean 3.67/5, SD 0.93).

Common challenges reported by survey participants includes the high message volume reported by 68.0% (87/128) of respondents, difficulty keeping up with discussions (61.7%, 79/128), and fear/anxiety in participating (43.8%, 56/128), with the latter significantly more associated with preclinical students (*p* < 0.05).

Additionally, visualization of the Likert-scale responses were performed centred around neutral to aid in interpreting the positive and negative attitudes of survey respondents. These Likert-scale graphs can be found within the Supplementary material (see Supplementary Figure 1 and Supplementary Figure 2; Tables 1–3).

**Table 1:** Participant demographic data (*N* = 128).

Characteristic	Participants, *n* (%)
Gender	
Female	96 (75.0)
Male	32 (25.0)
Year of study	
Year 2	42 (32.8)
Year 3	33 (25.8)
Year 4	15 (11.7)
Year 5	17 (13.3)
Year 6	14 (10.9)
Alumnus	7 (5.5)
Institutional clinical training	
Received no clinical training	75 (58.6)
Received clinical training	53 (41.4)
Age in years	
≤17	0 (0)
18–21	90 (70.3)
22–25	34 (26.6)
26–30	2 (1.6)
≥30	2 (1.6)

**Table 2:** Comparison of mean Likert scales are rated by responders from a scale of 1 to 5 by clinical experience status amongst MBRU Radiology Club members.

Variable	Overall mean (SD) (*N* = 128)	Preclinical student mean (SD) (*n* = 75)	Clinical student mean (SD) (*n* = 53)	*t* (*df*)	*p*	95% confidence interval	Cohen’s *d*
Group chat comfort (1 = strongly negative, 5 = strongly positive)							
General comfort using WhatsApp.	4.50 (0.96)	4.32 (1.09)	4.75 (0.65)	−2.82 (122.71)	0.006	[−0.74, −0.13]	−0.47
General comfort learning from WhatsApp.	3.88 (1.12)	3.79 (1.07)	4.02 (0.93)	−1.28 (126.00)	0.204	[−0.59, 0.13]	−0.23
Comfort learning from the Radiology Club WhatsApp group chat.	3.82 (1.01)	3.71 (1.05)	3.98 (0.93)	−1.53 (126.00)	0.129	[−0.63, 0.08]	−0.27
Group usage (1 = never, 5 = multiple times a day)							
General WhatsApp usage time.	4.56 (0.86)	4.55 (0.86)	4.57 (0.87)	−0.13 (126.00)	0.901	[−0.33, 0.29]	−0.02
Club group chat usage time.	3.1 (0.96)	3.08 (0.89)	3.11 (1.07)	−0.19 (126.00)	0.848	[−0.38, 0.31]	−0.03
Accessibility (1 = strongly disagree, 5 = strongly agree)							
The group resources (images, videos, discussions) are clear, high resolution, and can be downloaded and opened immediately when clicked on.	4.45 (0.63)	4.41 (0.66)	4.51 (0.58)	−0.86 (126.00)	0.394	[−0.32, 0.13]	−0.15
Effectiveness (1 = strongly disagree, 5 = strongly agree)							
The Radiology Club’s WhatsApp teaching methods are effective.	3.82 (0.86)	3.75 (0.89)	3.92 (0.83)	−1.15 (126.00)	0.253	[−0.48, 0.13]	−0.21
The Radiology Club’s WhatsApp group chat boosted my confidence in answering radiology-related questions during practical and theoretical lessons.	3.30 (1.19)	3.20 (1.13)	3.43 (1.26)	−1.10 (126.00)	0.273	[−0.66, 0.19]	−0.20
The Radiology Club’s WhatsApp group chat helped me in answering questions during in-house exams (e.g., written, OSCEs, case presentations).	3.35 (1.13)	3.33 (1.14)	3.38 (1.26)	−0.22 (126.00)	0.830	[−0.49, 0.36]	−0.04
The Radiology Club WhatsApp group enhanced my clinical performance and confidence in my rotation (e.g., interpreting images, case discussions).	--^*^	--^*^	3.70 (1.20)^a^	--^*^	--^*^	--^*^	--^*^
The Radiology Club’s WhatsApp group chat helped me prepare for my board exams (e.g., USMLE).	--^*^	--^*^	3.41 (0.91)^b^	--^*^	--^*^	--^*^	--^*^
Engagement (1 = strongly disagree, 5 = strongly agree)							
I feel comfortable participating in discussions on the WhatsApp group.	3.34 (1.05)	3.21 (1.00)	3.51 (1.09)	−1.59 (126.00)	0.115	[−0.67, 0.73]	−0.29
I feel that the WhatsApp group is a safe learning environment.	3.51 (1.09)	3.33 (1.11)	3.75 (1.04)	−2.18 (126.00)	0.031	[−0.80, −0.04]	−0.39
Participating in the Radiology Club WhatsApp group is enjoyable.	3.67 (0.93)	3.78 (0.90)	3.49 (0.95)	1.58 (126.00)	0.117	[−0.07, 0.65]	0.31
Validity (1 = strongly disagree, 5 = strongly agree)							
The Radiology Club WhatsApp group’s teaching methods and materials align with institutional learning outcomes.	4.31 (0.77)	4.37 (0.79)	4.23 (0.75)	1.06 (126.00)	0.290	[−0.13, 0.42]	0.19
The Radiology Club WhatsApp Group’s teaching methods and materials effectively help you assess your current level in relation to your college.	3.82 (0.99)	3.84 (0.95)	3.79 (1.06)	0.27 (126.00)	0.790	[−0.31, 0.40]	0.05
The Radiology Club WhatsApp group’s teaching methods and materials help you achieve your learning goals.	3.64 (1.10)	3.67 (1.00)	3.60 (1.23)	0.32 (126.00)	0.751	[−0.33, 0.46]	0.06
The information and resources provided by the Radiology Club WhatsApp Group is reliable and credible.	4.41 (0.76)	4.43 (0.74)	4.40 (0.79)	0.22 (126.00)	0.824	[−0.24, 0.30]	0.04

Scores were measured on a 5-point Likert scale (1 = strongly negative, 5 = strongly positive).

* Preclinical students (years 2 and 3) have not been exposed to clinical rotations, and are not eligible to sit for national or international board exams; thus, the analysis examining response difference between preclinical and clinical students was not performed for these metrics.

a *n* = 27.

b *n* = 22.

**Table 3:** Participant preferences reported by the club’s group chat participants (*N* = 128).

Category	Participants, *n* (%)
What platform features of the Radiology Club WhatsApp group do you find useful?	
Shared radiology cases/images	105 (82.0)
Annotated images with explanations	98 (76.6)
Faculty guidance and discussions	90 (70.3)
Peer collaboration and discussions	77 (60.2)
The ability to ask and clarify doubts instantly	62 (48.4)
Sharing and receiving resources like PDFs, links, and videos	50 (39.1)
Engaging with peers/socializing	48 (37.5)
Sending images/videos in high resolution (HD)	44 (34.4)
Accessibility to archived messages/images	41 (32.0)
Presenting cases you see	35 (27.3)
None of the above	0 (0.0)
Other	0 (0.0)
What educational features of the Radiology Club WhatsApp group do you find useful?	
Easy access to radiology cases anytime/anywhere	100 (78.1)
Exposure to a wide variety of cases beyond formal curriculum	93 (72.7)
Quick sharing of images and discussions	87 (68.0)
Encourages active learning and critical thinking	70 (54.7)
Guidance/insights from faculty or seniors	63 (49.2)
Builds confidence in interpreting radiological findings	62 (48.4)
Peer-to-peer learning and collaboration/networking	60 (46.9)
Convenient compared to formal platforms (email, LMS, etc.)	56 (43.8)
Helps in exam preparation / OSCE / clinical rotations	53 (41.4)
Immediate feedback and clarification from peers/seniors	53 (41.4)
Motivation to study radiology more regularly	50 (39.1)
Bridges the gap between theory and clinical practice	49 (38.3)
Gamification elements (e.g., leaderboard, quizzes, prizes)	43 (33.6)
Opportunity to ask questions without judgment	42 (32.8)
Fosters sense of community and belonging	39 (30.5)
Encourages concise communication of findings	39 (30.5)
Ability to edit and annotate on shared images	39 (30.5)
Increases interest in radiology as a career	35 (27.3)
None of the above	2 (1.6)
Other	0 (0.0)
What challenges do you face while using the WhatsApp group for radiology education?	
Overwhelming volume of messages	87 (68.0)
Difficulty keeping up with discussions	79 (61.7)
Fear of making mistakes in front of peers	56 (43.8)
General anxiety in participating	54 (42.2)
Difficulty finding/retaining older case information (no formal archive)	30 (23.4)
Inability to participate actively	27 (21.1)
Complexity of cases/discussions	25 (19.5)
Inconvenient case-discussion timings	25 (19.5)
Nothing in particular	13 (10.2)
Not enough faculty/senior input/supervision	6 (4.7)
Technical issues (limited internet/data access, crashing, etc.)	3 (2.3)
Other	2 (1.6)
What features or improvement to existing features would improve the WhatsApp group as an educational tool?	
Structured learning sessions (e.g., scheduled case discussions)	61 (47.7)
Better categorization or archiving of shared resources (e.g., folders/website for cases or topics)	59 (46.1)
Moderation to maintain focused discussions	49 (38.3)
More gamification elements	45 (35.2)
Integration with external educational tools (e.g., links to radiology reference platforms)	34 (26.6)
Establishing a safe environment for participants	34 (26.6)
Regular feedback from participants/faculty to improve group effectiveness	23 (18.0)
Nothing in particular	18 (14.1)
Enhanced group privacy and data security measures	15 (11.7)
Other	2 (1.6)

Note that participants can answer with multiple categories per question.

## 5 Discussion, lessons learned, and recommendations

### 5.1 Principal findings

This exploratory study investigated learner perceptions of a structured case-discussion framework used in a structured student-led, faculty-moderated online group chat for undergraduate radiological education. The study was conducted to assess perceived usefulness of the MBRU Radiology Club WhatsApp group chat amongst club members in the College of Medicine at the Mohammed Bin Rashid University of Medicine and Health Sciences (MBRU), Dubai, UAE. This cross-sectional study of 128 members of the MBRU Radiology Club found that students generally view the student-led and faculty-moderated instant-messaging platform and framework as highly accessible, multimedia-friendly, credible, and valid while moderately safe and effective as an adjunctive extra-curricular educational tool for radiology.

The patterns and quantitative results reported in this study suggest that learners may perceive value in an internet-based group chat with a structured, student-led, and faculty-moderated approach to image-based teaching via the IIIDDS (Indication, Imaging, Interpretation, Differentials, Discussion, and Safety-net) online group chat case presentation framework employed by the club.

### 5.2 Interpretation of findings

The Kirkpatrick model is a four-level framework for evaluating educational interventions across reaction, learning, behaviour, and results (42). The evaluation from this study aligns primarily with Kirkpatrick Level 1 (learner reaction) and aspects of Level 2 (self-reported learning/confidence), without objective performance measures (42, 44). The findings suggest that the educational value of the WhatsApp group may be driven by both the platform itself and the structured educational design embedded within the Radiology Club group chat. Particularly, the high ratings for image quality, annotation visuals, and faculty guidance indicates that learners may benefit most from elements that reduced cognitive load and ambiguity while possibly providing credibility, an element supported by the cognitive load theory (16). The IIIDDS structure may function as a scaffold that organizes and gives structure to an otherwise formless open discussion platform to promote coherent group discussions instead of fragmented individualistic interactions. By linking cases to clinical indication through addressing differential diagnoses, the framework may help learners engage with clinical reasoning processes in a guided manner that preclinical students may be exposed to at an earlier stage due to potential student engagement with the framework. The higher reported comfort levels and perceived safety among clinical students, compared with preclinical students, may reflect their greater familiarity with clinical-based case learning styles. In contrast, preclinical students may be limited by gaps in knowledge in specific topics; however, the inclusion of a “safety-net” stage within the IIIDDS model was designed to help mitigate anxiety for less experienced learners by normalizing uncertainty and encouraging questions when doubts arise. We also speculate that providing participation/partial points to incorrect answers may also reduce fear of making mistakes while normalizing uncertainty that arises from learning as described by Schneid et al. (45). Overall, the findings suggest that a structured educational design for online group chats may shape learners’ engagement and perceptions of learning value in online group settings. To mitigate information overload and improve retention in large group chat learning environments, future iterations may benefit from predictable scheduled structured sessions, weekly summaries, and case-archiving via a website.

### 5.3 Adaptability beyond radiology

The IIIDDS model was developed in the context of radiology’s image-rich environment; however, the framework may be adaptable to other clinical and non-radiological educational contexts. For non-radiology-based disciplines that rely on visual pattern recognition (for example, dermatology, ophthalmology, histopathology, point-of-care ultrasound, wound care, etc.), the *Imaging* step may still be relevant and may be representative of photographs, microscopy slides, or sonographic findings that all may still be annotated. For disciplines that are less image-based (e.g., electrocardiography, lab interpretation, pharmacology case problems, etc.), the *Imaging* step may be substituted for a *Findings* step, which may represent electrocardiogram tracings, lab panels, physical examination findings, patient history, etc. in image or text format, without any other alterations in the IIIDDS framework. The model may also be used in the context of other instant-messaging software, not just WhatsApp, if the instant-messaging software facilitates accessibility and ease of use in the context of multimedia sharing.

### 5.4 Comparison to the literature

The findings of this study are consistent with prior studies reporting the benefit of the WhatsApp and other instant-messaging platforms in enhancing engagement, accessibility, and perceived medical learning (8, 46, 47). Particularly, the benefits of WhatsApp group chats are reported in radiological teaching environments due to the accessibility of visually oriented message-sharing tools offered by the platform (13, 14). Previous literature has emphasized WhatsApp as a medical education tool for rapid communication, multimedia sharing, and peer interaction; however, these studies usually describe the instant-messaging platform used in an unstructured manner and not in the context of undergraduate medical students; thus, this study extends existing work by emphasizing the potential benefits of a standardized and repeatable case presentation framework possibly offered by the IIIDDS model in training future physicians, not just radiologists (8). This study further extends on prior work by potentially demonstrating that a standardized and repeatable case framework, like the IIIDDS model, may support structured interactions and learning processes in medical curriculum development (26, 48). The preference for annotated images and faculty moderation also aligns with earlier findings that demonstrate success with faculty-moderated models with visual learning elements improving medical learning (49–52). Additionally, the differences in reported comfort between preclinical and clinical students is represented by reports that mobile learning tools are perceived better once learners acquire sufficient clinical knowledge and exposure (53, 54). Moreover, it has been reported that structured frameworks, like the IIIDDS model, demonstrate improved learning outcomes through repeated and guided exposure to a variety of cases (55). A systematic review by Cook et al. supports the idea that structured learning designs facilitated by technology is what drives effective learning strategies in internet-based health education (56). This study adds to the literature by illustrating how learners perceived a structured learning model delivered through an online instant-messaging platform, contributing to the growing literature on messaging-based medical education.

### 5.5 Constraints

This study was a single-institution convenience sample of the MBRU Radiology Club members, potentially introducing sampling bias as members and survey participants are more likely to be motivated and interested in radiology. This cross-sectional study cannot establish that IIIDDS or WhatsApp produced any significant improvement in objective outcomes, like exam performance, as student performance was not measured. Leaderboards, club culture, and the presence of active faculty moderators may have altered perception despite anonymization of responses. A lack of a comparator/control group may also limit conclusions regarding the effectiveness of the WhatsApp platform or the IIIDDS model. Finally, although the survey employed the CHERRIES and TAM/TPB models, social desirability and recall biases may still exist. These limitations suggest caution in assuming the model will perform identically in different contexts.

### 5.6 Future directions

Future work should build beyond the exploratory and perception-based outcomes of the IIIDDS model via a prospective cohort or controlled design with objective learner outcomes (such as pre/post intervention knowledge scores, OSCE-style performance metrics, and longitudinal retention examination), ideally across multiple institutes/settings and other disciplines to help better generalizability. Iterative implementation of our model should evaluate practical refinements and participant highlights, such as scheduled sessions, structured weekly summaries, and searchable case archives to reduce message overload and promote safer learner environments.

## 6 Conclusion

Amongst undergraduate medical students, a student-led, faculty-moderated WhatsApp group chat using the IIIDDS case presentation framework was generally perceived as an engaging, accessible, reliable, and effective adjunct for radiology learning. The IIIDDS model may provide a structure that guides and organizes group chat-based case discussions, possibly supporting learners’ engagement with orientation, interpretation, and clinical reasoning processes. This model offers a potential framework for message-based case teaching which may possibly be expanded to fields other than radiology.

## Data Availability

The datasets presented in this article are not readily available because of institutional policies. Datasets generated or analysed during this study are available from the corresponding author on reasonable request. Requests to access the datasets should be directed to Rania Soued, rania.soued@dubaihealth.ae.
