# Supplementary material for: Design, implementation, and evaluation of IIIDDS: a structured WhatsApp case-discussion curriculum in undergraduate radiology education
Source: Front Med (Lausanne). 2026 Apr 10;13:1789785. doi: 10.3389/fmed.2026.1789785 (PMC13106052; doi:10.3389/fmed.2026.1789785)
Supplement: Supplementary file 2 [file Data_Sheet_1.PDF]

Supplementary Material S1

IIIDDS Replication Package: Presenter Template, Moderator Checklist, and Example Prompt Sequence

This toolkit is designed to support implementation of structured, case-based discussions in large instant-messaging group chats using the IIIDDS framework: Indications, Imaging (or Findings), Interpretation, Differentials, Discussion, and Safety-net. Replace bracketed text with your local details.

1. How to use this supplement

Use Sections 2–4 as a plug-and-play set of materials for running a single case discussion. The template assumes a student-led presenter overseen by a faculty moderator; adapt roles as needed for your context.

2. Roles and responsibilities

Presenter (learner/faculty)

- Curates a suitable teaching case and prepares de-identified images/media.
- Runs the discussion using the IIIDDS sequence and posts prompts at a steady pace.
- Guides the discussion and directs learner attention.
- Posts a brief end-of-case summary and key take-home points.

Academic lead (senior student/teaching assistant; optional)

- Checks academic accuracy, helps structure prompts, and anticipates common misconceptions.
- Supports the presenter during the live discussion (e.g., manages incoming questions, nudges pacing).

Faculty moderator (recommended)

- Reviews the case content before posting (accuracy, appropriateness, confidentiality).
- Provides clarifications during the discussion and reinforces key learning points.
- Ensures a respectful learning climate and reinforces patient-safety messaging.

3. Minimum confidentiality and de-identification checklist

- Remove all direct identifiers (name, identification code(s), date of birth, address, phone numbers, etc.).
- Avoid rare/unique contextual details that could indirectly identify a patient; generalize when possible.
- If using externally sourced images, confirm reuse permissions and cite the source in the post or summary.
- Obtain local approvals/permissions as required by your institution and platform policies.

4. Presenter template (copy/paste script)

Tip: Post one prompt at a time and allow a short window for replies (e.g. a minimum of 2-5 minutes) depending on group size and activity. Use a consistent tagging convention, such as "Case #___, Topic: ___, Modality: ___".

IIIDDS step	Goal	Prompt	Implementation tips
Pre-brief (before posting)	Ensure the case is ready, safe, and teachable.	What to prepare: 2–4 images (or Findings), final diagnosis, 3–5 learning points, and 1–2 references/resources.	Suggested post (not in chat): Run through the IIIDDS prompts and confirm pacing with your academic lead/moderator.
Indications	Anchor the case in a brief clinical context and ask learners to select an initial investigation.	"Case #___: [1–2 sentence vignette]. What is the most appropriate initial imaging/investigation?"	Encourage justification (e.g., "why CT vs X-ray?"). Optionally offer multiple-choice options to increase participation.
Imaging (or Findings)	Share the key image(s) or finding(s) and elicit a structured description.	"Here is the [modality/view]. What do you see? (Bonus: what additional views/tests would you request?)"	Share images in high resolution; use basic annotations only after initial responses to avoid giving away the answer.
Interpretation	Facilitate back-and-forth reasoning about salient findings and patterns.	"Let's interpret systematically: [ABC approach or modality-specific checklist]. What are the key abnormalities and likely mechanism?"	Acknowledge partial answers, ask targeted follow-ups ("Where is the abnormality?" "What sign supports that?").
Differentials	Generate and refine a differential diagnosis using clinical + imaging data.	"List your top 3 differentials and one feature that supports each. What extra information/investigation would help narrow it?"	Help rule in/out by prompting discriminating features; keep the list short and high-yield.
Discussion	Reveal the diagnosis and connect it to management, communication, and safety implications.	"Diagnosis: ___. How would you manage next? What is the key message you would communicate to the team/patient?"	Include a brief management algorithm or 'next steps' list; highlight red flags and time-critical actions.
Safety-net	Normalize uncertainty, invite questions, and close with a concise summary.	"Any questions or uncertainties? Key take-home points: (1)… (2)… (3)… Resources: [link/refs]."	Consider awarding partial credit for attempts to reduce fear of being wrong; encourage learners to DM questions if preferred.

5. Faculty moderator checklist

Before the session

- Confirm the case is fully de-identified and appropriate for the group's level.
- Verify the final diagnosis and key learning points; identify likely misconceptions.
- Confirm any externally sourced images/media have reuse permission or are openly licensed.
- Agree on timing (start/end) and escalation plan if clinical advice questions arise.

During the session

- Maintain psychological safety: reinforce that partial answers and questions are welcome.
- Step in to correct misinformation promptly and respectfully.
- Add brief "expert pearl" comments at 1–2 key moments (avoid dominating the discussion).
- Ensure patient-safety framing when discussing management and communication.

After the session

- Encourage a concise final summary (diagnosis, key findings, differentials, management takeaways).
- If your group archives cases, confirm the summary and images are stored without identifiers.
- Suggest one follow-up resource (guideline, review article, short video) for reinforcement.

6. Example prompt sequence (de-identified template)

Below is an example of how a single case might be posted. Replace details and images with your own de-identified materials. The sequence is intentionally short to fit typical group-chat attention spans.

Presenter (tag): Case #12 | Topic: Trauma | Modality: X-ray/CT

Presenter (Indications): A 54-year-old man presents after a fall with severe leg pain and deformity. What is the most appropriate initial imaging/investigation?

Presenter: [Allow replies; optionally add: "Why that modality?"]

Presenter (Imaging): Here is the initial image: [attach Image 1]. What are the key findings? (Bonus: what additional view/test would you request?)

Presenter: [After replies, attach Image 2 or add an annotation to highlight the key abnormality.]

Presenter (Interpretation): Let's interpret systematically: location, alignment, joint involvement. What pattern of injury is this and what complications should we consider?

Presenter (Differentials): List your top 2–3 differentials (or injury patterns) and one feature that supports each. What additional information would help confirm?

Presenter (Discussion): Diagnosis: [final diagnosis]. What are the immediate management priorities and what would you communicate to the receiving trauma team?

Faculty moderator (pearl): [1–2 sentences: key imaging sign + a practical pitfall to avoid.]

Presenter (Safety-net): Any questions? Take-home points: (1) … (2) … (3) … Resources: [1–2 links/citations].

7. Optional: simple archiving and tagging (to reduce information overload)

If participation drops due to message volume, consider adopting lightweight information retention practices:

- Schedule cases at predictable times and discourage off-topic posting during teaching windows.
- Use consistent case tags (case number/date/topic/modality) to improve searchability.
- Post a brief end-of-case summary and store summaries in a shared index (e.g., spreadsheet with links to de-identified files).

End of Supplementary Material S1
