# Supplementary figures and images for: Design, implementation, and evaluation of IIIDDS: a structured WhatsApp case-discussion curriculum in undergraduate radiology education
Source: Front Med (Lausanne). 2026 Apr 10;13:1789785. doi: 10.3389/fmed.2026.1789785 (PMC13106052; doi:10.3389/fmed.2026.1789785)

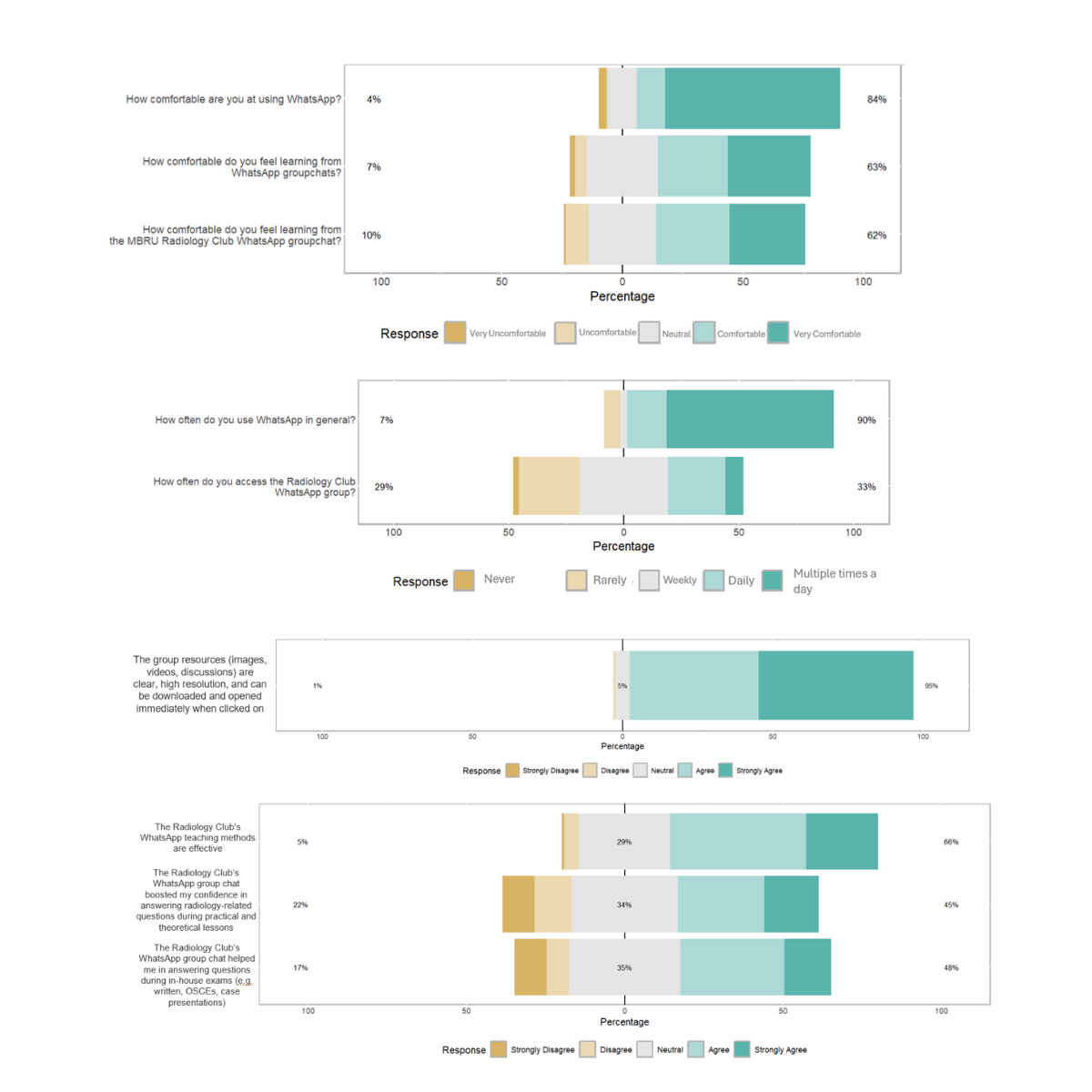

Supplement: Supplementary file 3 [file Image_1.PNG]

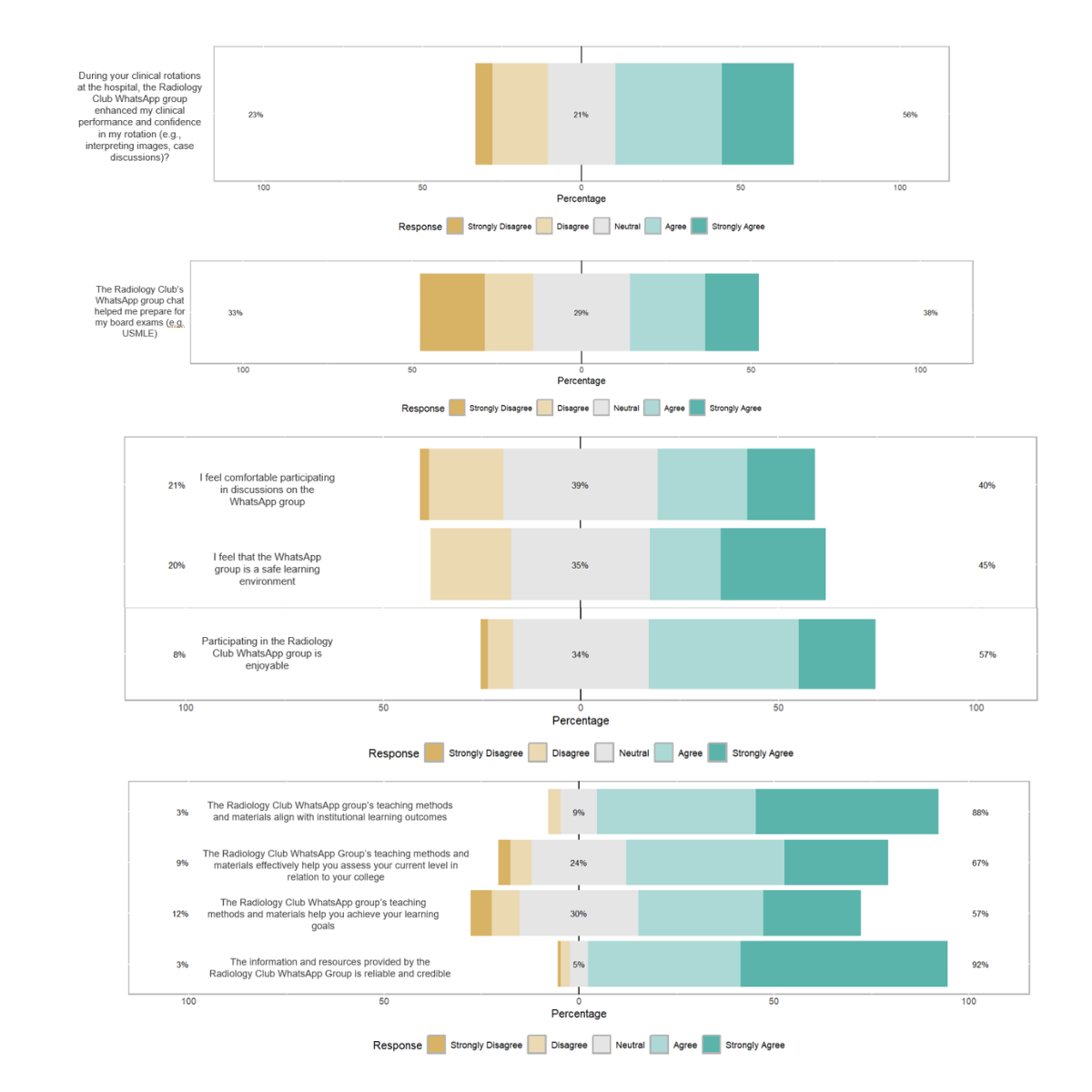

Supplement: Supplementary file 4 [file Image_2.PNG]
